# Comparative phylogeographic patterns and processes of the incipient brown seaweeds *Sargassum polycystum* and *S. plagiophyllum* around the Thai-Malay Peninsula

**DOI:** 10.3389/fpls.2025.1673650

**Published:** 2025-09-17

**Authors:** Tong-Yun Zhang, Qi-Qi Wang, Zhong-Min Sun, Stefano G. A. Draisma, Zi-Min Hu

**Affiliations:** ^1^ School of Ocean, Yantai University, Yantai, China; ^2^ Laboratory of Marine Organism Taxonomy & Phylogeny, Institute of Oceanology, Chinese Academy of Sciences, Qingdao, China; ^3^ Excellence Center for Biodiversity of Peninsular Thailand, Faculty of Science, Prince of Songkla University, Hat Yai, Songkhla, Thailand

**Keywords:** genetic admixture, long-distance dispersal, ocean current, incipient speciation, phylogeographic diversity, sea-level fluctuation

## Abstract

Incipient (recently-evolved) species are morphologically distinct and have a relatively short evolutionary history from a common ancestor, yet comparative phylogeographic patterns of incipient seaweed species have seldom been explored. Here, we created 339 mitochondrial *cox*1, 339 *cox*3, 339 *cox*1+*cox*3 and 286 nuclear ITS2 sequences for *Sargassum plagiophyllum* (10 populations) and 326 *cox*1, 336 *cox*3, 310 *cox*1+*cox*3 and 341 ITS2 sequences for *S. polycystum* (14 populations) around the Thai-Malay Peninsula (TMP), with the aim to explore potential drivers in shaping population genetic structuring and diversity over space, including a phylogeographic signature of incipient speciation. Comparative analysis showed that the two *Sargassum* species around the TMP diverged from their most recent common ancestor at *c*. 0.17 Mya, followed by a demographic expansion at *c*. 0.015–0.060 Mya. Oceanic currents drove contemporary continuous south-to-north gene flow in the Malacca Strait, leading to most genetic variation partitioned within populations and among populations within groups. Interestingly, the incipient *S. polycystum* and *S. plagiophyllum* shared their most common haplotypes/ribotypes, and mitochondrial datasets revealed much higher phylogeographic diversity in *S. polycystum* than in *S. plagiophyllum*. These results imply that the late Quaternary sea-level fluctuations and contemporary oceanic currents co-contributed to population genetic structuring and demographic histories of *S. polycystum* and *S. plagiophyllum* around the TMP. Importantly, comparative phylogeographic analysis of *S. polycystum* and *S. plagiophyllum* shed lights on the existence of a few separate late Pleistocene glacial refugia, such as the Andaman Sea for *S. plagiophyllum* and the northern Malacca Strait for *S. polycystum*, particularly the revealing of important signatures of their incipient speciation.

## Introduction

The Sunda shelf is located at the Indo-Pacific convergence region encompassing the Thai-Malay Peninsula (TMP) and the islands of Sumatra, Java, Borneo. Glacial and interglacial cycles during the Pleistocene epoch (1.8–0.012 million years ago (Mya)) caused the Sunda shelf to experience severe sea level fluctuations ranging from -120 m to 6 m compared to the present-day sea level, significantly altering the land bridge structure and waterbody connectivity in the Indo-Pacific convergence region (e.g. the disruption and reconnection between the east and west sides of the TMP, Hall, 1998; Voris, 2000). These geological configurations driven by ice cycles enabled the Sunda shelf to be an important marine biodiversity hotpot (Woodruff, 2010; Hall, 2012; Morley, 2012).

**Figure 1:**
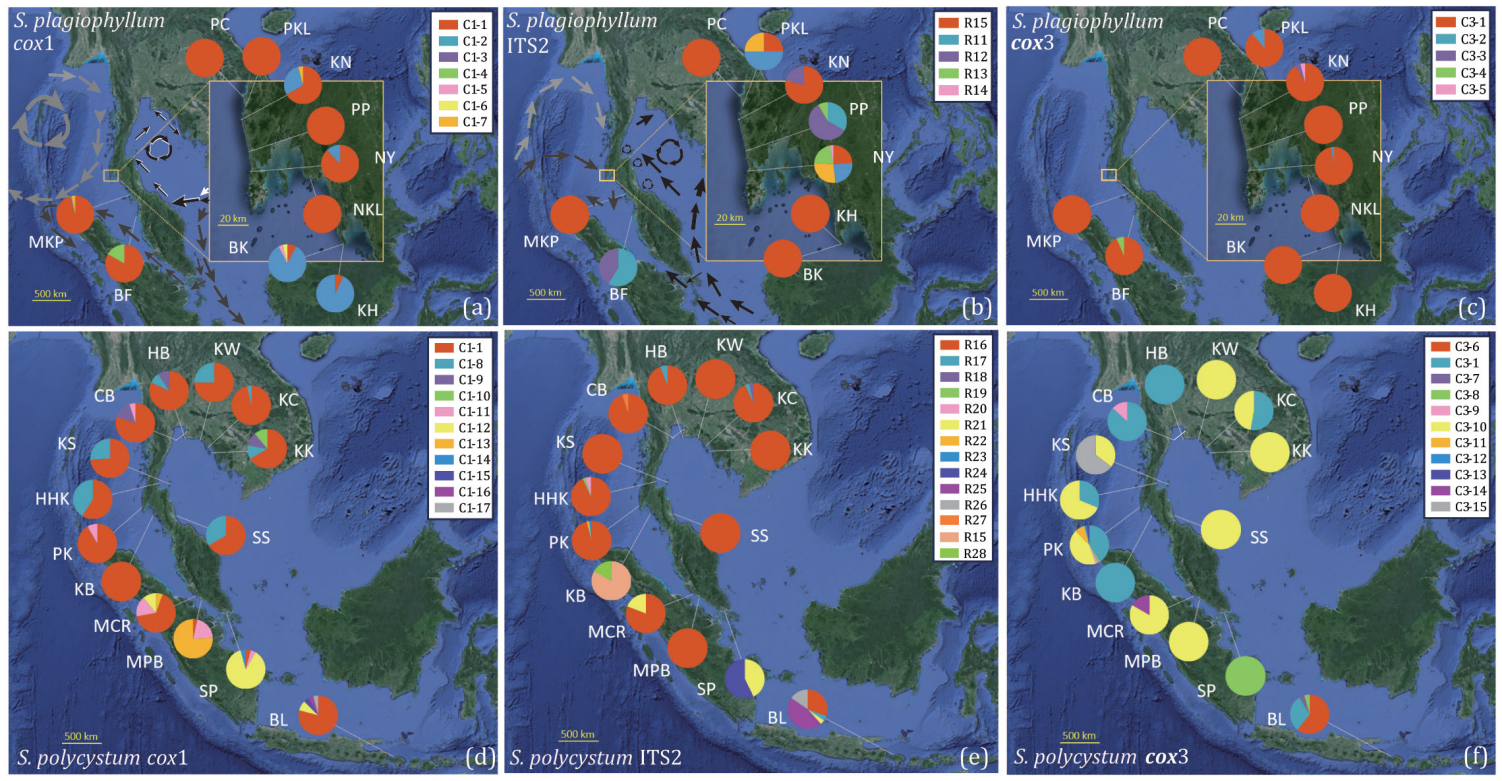
Geographical distribution and relative frequency of *cox*1 and *cox*3 haplotypes and ITS2 ribotypes for *S. plagiophyllum* **(A–C)** and *S. polycystum* **(D–F)**. The ocean currents are plotted under the prevailing northeast **(A)** and southwest monsoons **(B)** (modified from Chan et al., 2022). a: Gray arrows indicate the clockwise circulation in the Andaman Sea during the northeast monsoon; dark gray arrows indicate the currents from the South China Sea flow through the Singapore Strait into the Malacca Strait and reach the northern part of Sumatra Island; white and black arrows indicate currents flowing from the Western Pacific and the Philippines into the Gulf of Thailand. b: light gray arrows indicate ocean currents from the Indian Ocean passing through the Andaman Islands and flowing towards the north coast of the Andaman Sea during the southwest monsoon; dark gray arrows indicate ocean currents from the Indian Ocean passing through the south of the Nicobar Islands and flowing towards the south of the Andaman Sea; black arrows indicate ocean currents from the Java Sea flowing northward towards Singapore and then along the east coast of the Malay Peninsula, and ultimately into the Gulf of Thailand.

The TMP forms a natural biogeographical barrier between the Andaman Sea-Malacca Strait and the Gulf of Thailand that influences species distributions and genetic variability by hindering dispersal between its east and west coast (Gaither et al., 2010). Seasonal monsoons influence ocean surface currents significantly (Buranapratheprat, 2008), potentially driving species’ long-distance dispersal and population genetic structuring (Wee et al., 2014; 2020; Chan et al., 2022). During the northeast monsoon (December-February) a clockwise circulation forms in the Gulf of Thailand along with a clockwise flow in the Andaman Sea (**Figure 1A**). While during the southwest monsoon (June-August), some small-scale counterclockwise vortices develop along the west coast of the Gulf of Thailand (**Figure 1B**), reversing the direction of seawater flow through the Malacca Strait (Sojisuporn et al., 2010). The long-term interplay between ocean currents and paleoenvironmental changes considerably impacted speciation, biogeographic processes and population structuring of pelagic vertebrates and invertebrates around the TMP (Woodruff, 2010; Chan et al., 2022). Such a contribution is thus hypothesized to occur in marine sessile species such as macroalgae (Chan et al., 2013; Bulan et al., 2022).

The mapping of comparative phylogeographic structuring and diversity patterns served as a powerful tool for investigating evolutionary processes in a certain region (Bermingham and Moritz, 2010). Previous comparative studies exploring genetic diversity and phylogeographic patterns around the TMP, primarily focused on marine animals (Briggs, 2005; Reaka et al., 2008; Carpenter et al., 2011; Mirams et al., 2011). Recently, Chan et al. (2022) compared the distribution of intertidal *Tetraclitas* Schumacher barnacle species across the east and west sides of the TMP, concluding that geological vicariance during the Pleistocene glaciations and circulation-driven long-distance dispersal caused the predominant distributions of *T*. *singaporensis* Chan, Tsang & Chu in the Andaman Sea and *T*. *squamosa* Bruguière in the Gulf of Thailand, respectively. Seaweeds around the TMP also received increasing interest in the past decade, although in a non-comparative phylogeographic context. For example, the brown seaweed *Padina boryana* Thivy was found to be represented by two distinct lineages around the TMP of which one was restricted to the northern Gulf of Thailand (Wichachucherd et al., 2014). Muangmai et al. (2023) further detected a possible genetic break between populations of the red seaweed *Gracilaria salicornia* (C.Agardh) E.Y.Dawson in the Andaman Sea. More recently, Wang et al. (2025) found low phylogeographic diversity and shallow population structuring in the green alga *Halimeda macroloba* Decaisne around the TMP. These single-species based phylogeographic studies largely enhanced our understanding of how geological events and ocean currents interacted to affect geographic distribution and lineage structuring around the TMP. However, comparative phylogeographies of seaweeds, particularly for species originated in the mid-late Pleistocene (*c*. 0.8–0.1 Mya), are still poorly explored (e.g. Guillemin et al., 2018; Huanel et al., 2024).

Species of *Sargassum* C. Agardh, the most species-rich genus within the order Fucales (Phaeophyceae), serve as foundation species by providing essential ecological roles for other marine species (Faga and Gurgel, 2024). *Sargassum* has a diplontic life cycle, and the fertilized eggs remain attached to the receptacle for 2–3 days after fertilization (Draisma et al., 2010). The detached zygotes exhibit limited dispersal capabilities (<1 m) (Kendrick and Walker, 1995). However, *Sargassum* thalli exhibit gas-filled vesicles (aerocysts) which provide buoyancy for detached thalli (Komatsu et al., 2007; Mizuno et al., 2014), facilitating long-distance dispersal. This can partially explain why the genus *Sargrassum* currently has a cosmopolitan distribution spanning the Atlantic, Pacific, and Indian Oceans (Guiry and Guiry, 2025), whereas fucalean genera without aerocysts usually have a more limited distribution range (Draisma et al., 2010). The origination and diversification of the genus *Sargassum* was estimated to occur approximately at 4.3 Mya, and the longer history of diversification relative to other marine realms gave rise to higher species richness in the temperate northern Pacific (Yip et al., 2020).

*Sargassum polycystum* C. Agardh and *S. plagiophyllum* C. Agardh belong to the section *Polycystae* Mattio & Payri of the subgenus *Sargassum* and are prevalent throughout the Indo-Pacific convergence region (Soe-Htun et al., 2012). Geographically, *S. polycystum* occurs throughout the tropical Indo-West Pacific from Africa in the west to Tonga in the east (Guiry and Guiry, 2025). The distribution range of *S. plagiophyllum* is much smaller and stretches from India in the west to Guangxi (China) and Queensland (Australia) in the east (Guiry and Guiry, 2025). *Sargassum polycystum* and *S. plagiophyllum* may occur in sympatry (Soe-Htun et al., 2012; Stankovic et al., 2022). The two species can be distinguished by morphological differences such as the secondary holdfasts, with *S. plagiophyllum* forming cauline leaves, whereas *S. polycystum* developing primary branches (Kantachumpoo et al., 2015). In addition, the main branches of *S. plagiophyllum* are smooth, whereas they are distinctly muricate in *S. polycystum* (Soe-Htun et al., 2012). *Sargassum polycystum* and *S. plagiophyllum* have been calibrated to originate from a common ancestor at *c*. 0.4 Mya (95% HPD interval: 0.1–0.8 Mya, Yip et al., 2020), and their evolutionary history and population genetic structuring are likely associated with the late Pleistocene glacial fluctuations (e.g. the southwest of Hainan Island, China served as a glacial refugium for the survival of ancestral relics of *S. polycystum*, Hu et al., 2018) and contemporary marine conditions (Lin et al., 2024). Organellar-genome scale calibrations further revealed their divergence at *c*. 0.1–0.5 Mya (Zhang et al., 2022). These lines of evidence indicate that *S. polycystum* and *S. plagiophyllum* are recently-evolved (incipient) species, allowing us to hypothesize that kinds of organellar genes between them may share the same sequence composition. Therefore, comparing their phylogeographic patterns and processes of these two *Sargassum* species can bridge the knowledge gap between evolutionary processes and genetic signatures of the incipient speciation in seaweeds.

In this study, we sequenced the mitochondrial cytochrome *c* oxidase subunits I and III (*cox*1 and *cox*3) and the nuclear internal transcribed spacer 2 (ITS2) of *S. polycystum* and *S. plagiophyllum* populations around the TMP. Our aims are to test two hypotheses: i) the TMP acted as a geographical barrier contributing to population genetic connectivity of *S. polycystum*; ii) the late Pleistocene ice ages and contemporary ocean currents shaped the phylogeographic structuring and history of two *Sargassum* species around the TMP. By comparing population genetic variation and biogeographic patterns of two *Sargassum* species, we are also interested in exploring DNA-based phylogeographic signatures of recently-evolved species in the subgenus *Sargassum*. The results can offer valuable insights for managing and conserving *Sargassum* resources around the TMP, providing new knowledge to understand speciation, diversification and evolution in the Sunda Shelf.

## Materials and methods

### Sample collection, DNA extraction, amplification, and sequencing

*Sargassum plagiophyllum* has never been reported from the east coast of the TMP, and on the west coast Penang Island harbours the southmost known population. On the Thai west coast, *S. polycystum* is rare and only a single small population was found in the lower intertidal in Phuket (PK), where *S. plagiophyllum* also was collected (NY) (**Table 1**). This is the only site around the TMP where the two species grow in sympatry, but locally they were separated in water depth. Based on the predicted distribution range of two *Sargassum* species (Stankovic et al., 2022), *S*. *plagiophyllum* from 10 localities and *S*. *polycystum* from 14 localities were sampled respectively (**Table 1**, **Figure 1**). At each locality, 3–5 cm of the youngest branch (3–97 individuals per locality (**Table 1**), including leaves, aerocysts, and receptacles) that appeared without epiphytes were cut and preserved in silica gel. All populations sampled in the Gulf of Thailand grew in the shallow subtidal, whereas those collected on the west coast grew in the intertidal. The collection of *Sargassum* samples in the field complied with the national legislation of Thailand, Malaysia, Singapore and Indonesia and international guidelines. Dr. Draisma Stefano undertook the formal identification of *S. polycystum* and *S. plagiophyllum*, and all voucher specimens were deposited in Department of Marine Science, Ocean School, Yantai University.

**Table 1:** Population genetic diversity indices of *Sargassum polycystum* and *S. plagiophyllum* in the Thai-Malay Peninsula based on mitochondrial *cox*1, *cox*3, *cox*1 + *cox*3 and nuclear ITS2.

Ecoregion	Sampling location		*Cox*1			*Cox*3			*Cox*1+*Cox*3			ITS2		
		Code	*n*/*N_h_*	*h*	*π* (×10^-2^)	*n*/*N_h_*	*h*	*π* (×10^-2^)	*n*/*N_h_*	*h*	*π* (×10^-2^)	*n*/*N_h_*	*h*	*π* (×10^-2^)
	*Sargassum plagiophyllum*													
I	Pakarang Cape, Khao Lak, Phang-nga, Thailand	PC	5/1	0.000	0.000	6/1	0.000	0.000	5/1	0.000	0.000	6/1	0.000	0.000
I	Khao Lak beach, Phang-nga, Thailand	PKL	9/1	0.000	0.000	9/2	0.222	0.036	9/2	0.222	0.019	8/3	0.714	0.944
I	Khao NaYak, Phang-nga, Thailand	KN	24/3	0.489	0.090	24/3	0.163	0.027	24/5	0.529	0.057	19/2	0.351	0.632
I	Pilai beach, Phang-nga, Thailand	PP	13/1	0.000	0.000	13/1	0.000	0.000	13/1	0.000	0.000	12/3	0.591	0.196
I	NaiYang beach, Phuket, Thailand	NY	41/2	0.220	0.038	41/2	0.049	0.008	41/3	0.262	0.023	33/5	0.786	0.919
I	Noppharat Thara beach, AoNang, Krabi, Thailand	NKL	35/1	0.000	0.000	35/1	0.000	0.000	35/1	0.000	0.000			
I	Bakantiang beach, Koh Lanta Yai (w), Thailand	BK	26/4	0.286	0.053	26/1	0.000	0.000	26/4	0.286	0.025	24/1	0.000	0.000
I	Khlong Hin, Koh Lanta Yai (w), Thailand	KH	59/2	0.129	0.023	59/1	0.000	0.000	59/2	0.129	0.011	58/1	0.000	0.000
I	Mu Ko Phetra, Satun, Thailand	MKP	97/3	0.080	0.014	97/1	0.000	0.000	97/3	0.080	0.007	97/1	0.000	0.000
II	Batu Ferringhi, Penang, Malaysia	BF	30/2	0.287	0.050	30/2	0.129	0.021	30/3	0.393	0.035	29/2	0.502	0.151
	*Sargassum polycystum*													
III	KohKut, Trat, Thailand	KK	9/4	0.583	0.156	9/1	0.000	0.000	7/4	0.714	0.096	11/1	0.000	0.000
III	Koh Chang, Trat, Thailand	KC	29/2	0.069	0.012	36/2	0.513	0.249	29/3	0.530	0.133	29/3	0.136	0.042
III	Kung Wiman beach, Chantha Buri, Thailand	KW	24/2	0.391	0.069	24/1	0.000	0.000	24/2	0.391	0.033	24/1	0.000	0.000
III	Haad Tien, Chonburi, Thailand	HB	11/3	0.346	0.096	12/1	0.000	0.000	7/3	0.524	0.072	17/2	0.118	0.036
III	Sattahip, Chonburi, Thailand	CB	20/3	0.353	0.106	23/2	0.237	0.038	20/4	0.558	0.074	20/2	0.100	0.061
III	Had Hin Kob, Chum Porn, Thailand	HHK	19/2	0.409	0.072	20/2	0.100	0.016	17/2	0.441	0.037	19/1	0.000	0.000
III	KohSamui, Surat Thani, Thailand	KS	44/2	0.495	0.087	48/2	0.439	0.213	43/4	0.710	0.146	44/3	0.132	0.041
III	Bo Dan, Sathing Phra, Songkhla, Thailand	SS	24/2	0.464	0.081	24/1	0.000	0.000	24/2	0.464	0.039	24/1	0.000	0.000
II	St John Island Port, Singapore	SP	24/4	0.239	0.083	18/1	0.000	0.000	18/4	0.314	0.052	21/2	0.514	0.156
IV	Bali Island, Indonesia	BL	24/5	0.377	0.085	23/4	0.557	0.202	23/8	0.739	0.148	26/5	0.692	0.877
II	Pulau Besar (W), Malacca, Malaysia	MPB	29/3	0.394	0.079	29/1	0.000	0.000	29/3	0.394	0.038	30/1	0.000	0.000
II	Cape Rachado, Malacca, Malaysia	MCR	18/4	0.542	0.152	18/2	0.294	0.190	18/4	0.542	0.172	21/2	0.324	0.098
I	Lanta Island, Krabi, Thailand	KB	3/1	0.000	0.000	3/1	0.000	0.000	3/1	0.000	0.000	6/2	0.333	0.010
I	NaiYang beach, Phuket, Thailand	PK	48/2	0.156	0.027	49/7	0.655	0.329	48/7	0.685	0.182	49/3	0.081	0.037

*n*, number of sequences; *N_h_*, number of haplotypes; *h*, haplotype/ribotype diversity; *π*, nucleotide diversity

I: the Andaman Sea Coral Coast ecoregion; II: the Malacca Strait ecoregion; III: the Gulf of Thailand ecoregion; IV: Lesser Sunda ecoregion of the Western Coral Triangle

Total genomic DNA was extracted from *c*. 0.3 g dried leaves using the FastPure Plant DNA Isolation Mini Kit (Vazyme Biotech Co., Ltd., Nanjing, China), following the manufacturer’s instructions. Mitochondrial *cox*1 and *cox*3, and nuclear ITS2 were selected as target markers according to previous phylogeographic studies of *Sargassum* (Hu et al., 2011; Li et al., 2017; Hu et al., 2018; Liang et al., 2022). The primer sets GazF2 and GazR2 (Lane et al., 2007), trnY-P1 and cox3-P2 (Kogishi et al., 2010) and 5.8S-BF and 25BR-2 (Yoshida et al., 2000) were utilized to amplify *cox*1, *cox*3 and ITS2, respectively (**Supplementary Table S1**). PCR protocols and purifications were conducted following Hu et al. (2018). PCR products were examined through 1% agarose gel electrophoresis and sequenced in both directions using the Applied Biosystems™ 3730XL at Bioengineering Co., Ltd. (Shanghai, China), using the same primer sets as PCR amplification.

### Genetic diversity and population structuring

*Cox*1, *cox*3 and ITS2 sequences were aligned and trimmed using MEGA v10.1.8 (Kumar et al., 2016). Mitochondrial haplotypes (*cox*1, *cox*3) and nuclear ribotypes (ITS2) were identified for both datasets of *S. plagiophyllum* and *S. polycystum* using DnaSP v5.10 (Librado and Rozas et al., 2009). Genetic diversity indices, including haplotype number (*N_h_*), haplotype diversity (*h*) and nucleotide diversity (*π*), were calculated using Arlequin v3.5 (Excoffier and Lischer, 2010).

Analysis of molecular variation (AMOVA) were conducted to partition genetic variation at different spatial scales. *Sargassum plagiophyllum* populations were categorized into two groups following the Marine Ecoregions of the World bioregionalization scheme proposed by Spalding et al. (2007): the Andaman Sea Coral Coast ecoregion (PC, PKL, KN, PP, NY, NKL, BK, KH, and MKP) and the Malacca Strait ecoregion (BF) (**Figure 1**). *Sargassum polycystum* populations were divided into three groups: the Gulf of Thailand ecoregion (KK, KC, KW, CB, HHK, KS, HB and SS which both were washed up), the Malacca Strait ecoregion (SP, MPB, and MCR), and the Andaman Sea Coral Coast ecoregion (PK, KB) (**Figure 1**). Population-level pairwise F_ST_ were estimated with Arlequin.

Subtle intraspecific genetic structuring between *S. plagiophyllum* and *S. polycystum* was performed using Structure v2.3.4 (Pritchard et al., 2000) based on an admixture model. For each dataset, five independent runs were performed for each value of K (number of clusters) from 1 to 5, based on the produced haplotype/ribotype networks and phylogenetic trees (see results below). The MCMC was set to 5×10^7^ iterations with a burn-in of 5×10^5^ iterations. The online tool Structure Selector (https://lmme.ac.cn/StructureSelector/) was used to calculate LnP(K) and ΔK (Evanno et al., 2005) to determine the optimal K value (**Supplementary Figure S1**) and draw clustering plots. *Cox*1 and *cox*3 datasets were also concatenated using MEGA to construct haplotype networks as well as to examine the consistence of population genetic structuring between *S. plagiophyllum* and *S. polycystum*.

### Demographic history and spatial migration

Bayesian skyline plots (BSPs) were conducted with BEAST v1.10.4 (Suchard et al., 2018) to assess historical changes in effective population size for each of the two species. An uncorrelated lognormal relaxed molecular clock was applied, with tree priors set to coalescent Bayesian skyline. The MCMC chains were run for 2×10^8^ iterations, with sampling conducted every 15,000 generations, and the first 15,000 iterations discarded as burn-in. Convergence of the treeset files was checked using Tracer v1.7.2 (Effective Sample Size (ESS) > 200) to generate a demographic trend chart.

Population-level migration driven by oceanic currents was estimated using Migrate-n v5.0.4 (Beerli and Felsenstein, 2001) for each dataset. The effective population size (θ = xN_e_μ, where N_e_ represents the effective population size, μ is the mutation rate per generation, and x is typically 4 for nuclear data and 1 for mitochondrial data) and the migration rate (M = m/μ, where m denotes the migration rate per generation and μ is the mutation rate) were substituted into the formula (Nm =θM/x) to calculate the effective number of migrants. The Monte Carlo Markov Chain (MCMC) was set to 100,000 iterations with a burn-in of 25,000 iterations, while heating parameters were configured as 1.0, 1.5, 3.0, and 1.0 under a static scheme.

### Haplotype/ribotype network and molecular dating

To detect the relationships between the newly identified haplotypes/ribotypes around the TMP and the hypothesized late-Pleistocene glacial survived ancestral genotypes (Hu et al., 2018), 15 *cox*1 haplotypes, 12 *cox*3 haplotypes and 11 ITS2 ribotypes of *S. polycystum* from Hainan and Guangxi provinces, China (Hainan-Guangxi) were retrieved. The network diagrams of all combined haplotypes and ribotypes were created with a median-joining method using Network v10.2.0.0 (Bandelt et al., 1999). In addition, all *cox*1 and *cox*3 haplotypes and ITS2 genotypes were pooled together, respectively to examine if there are shared genotypes between *S. polycystum* and *S. plagiophyllum* as well as among the Andaman Sea, the Gulf of Thailand, the Malacca Strait and Hainan-Guangxi.

For each combined haplotype/ribotype dataset, Modelfinder (Kalyaanamoorthy et al., 2017) was used to determine the optimal nucleotide substitution model under Bayesian information criteria (BIC) (*cox*1: HKY+F+I, I=0.835; *cox*3: HKY+F+G4, G=0.107; ITS2: HKY+F). Beast v1.10.4 was used to calculate the intraspecific level of divergence times, selecting an uncorrelated lognormal relaxed molecular clock with tree priority set to the Yule model. Node time was set as 0.4 Mya (95% HPD: 0.1–0.8 Mya, Yip et al., 2020) for the Section *Polycystae* and 1.5 Mya (95% HPD: 0.6–2.6 Mya) for the Section *Binderiana* (Yip et al., 2020). *Sargassum aquifolium* (GenBank accession no. *cox*1/*cox*3: NC_033408, ITS2: HF572039) and *S. patens* (*cox*1/*cox*3: NC_052831, ITS2: KY935432) in the Section *Binderiana* were chosen as outgroups. The MCMC chains were run for 5×10^8^ iterations, sampled every 5×10^4^ generations, and the first 5×10^4^ iterations discarded as burn-in. Tracer v1.7.2 was used to check convergence (ESS > 200). The topological structure and divergence times of the lineages were viewed using Figtree v1.4.4 (http://tree.bio.ed.ac.uk/software/figtree/).

## Results

### Population genetic diversity and variation

We obtained 339 *cox*1 (570 bp), 339 *cox*3 (618 bp), 339 *cox*1+*cox*3 (1188 bp), and 286 ITS2 (335 bp) sequences from 10 *S. plagiophyllum* populations (**Table 1**), which contained 6, 4, 10, and 8 variable sites, respectively. *Cox*1 and *cox*3 identified 7 and 5 haplotypes, respectively, with C1-1 (**Figure 1A**) and C3-1 (**Figure 1C**) as the most abundant and widely distributed. ITS2 identified 5 ribotypes, with R15 as the most abundant (**Figure 1B**). For *S. polycystum*, we obtained 326 *cox*1 (570 bp), 336 *cox*3 (618 bp), 310 *cox*1 + *cox*3 (1188 bp), and 341 ITS2 (335 bp) sequences from 14 populations (**Table 1**). The four datasets contained 9, 15, 22, and 14 variable sites, respectively. *Cox*1 and *cox*3 each identified 11 haplotypes, with C1-1 (**Figure 1D**) and C3-10 (**Figure 1F**) as the most abundant, and ITS2 identified 14 ribotypes, with R16 as the most common/frequent (**Figure 1E**). Concatenated *cox*1+*cox*3 yielded 11 and 24 haplotypes for *S. plagiophyllum* (**Supplementary Figure S2A**) and *S. polycystum* (**Supplementary Figure S2b**), respectively.

For *S. plagiophyllum*, *cox*1, *cox*3, and *cox*1+*cox*3 consistently identified a few site-specific haplotypes in population BF from Penang, Malaysia (**Figures 1A, C**, **S2A**). They also revealed relatively high haplotype and nucleotide diversity in the populations BF (Penang) and PKL and KN from Phang-Nga, Thailand (*cox*1+*cox*3: *h*=0.222–0.529; *π*=0.00019–0.00057, **Table 1**). ITS2 revealed much higher ribotype and nucleotide diversity in the populations PKL and NY (Phuket) from Thailand (*h*=0.714–0.786; *π*=0.00919–0.00944, **Table 1**). Other populations, particularly the PC (Phang-Nga), NKL (Krabi) and MKP (Satun) from in Thailand exhibited the lowest genetic diversity (*h*=0.000; *π*=0.00000, **Table 1**).

For *S. polycystum*, *cox*1, *cox*3, *cox*1+*cox*3, and ITS2 consistently identified site-specific rich haplotypes/ribotypes in population BL from Bali, Indonesia (**Figures 1D–F**, **S2B**). This enabled BL to harbour higher haplotype and nucleotide diversity (*cox*3: *h*=0.557; *π*=0.00202; *cox*1+*cox*3: *h*=0.739; *π*=0.00148) than other populations (*cox*3: the mean *h*=0.172; the mean *π*=0.00080; *cox*1+*cox*3: the *h*=0.482; the mean *π*=0.00083, **Table 1**). Except the KB, *cox*1 detected high level of genetic variation in Thai populations (*h*=0.069–0.583; *π*=0.00012–0.00156), whereas *cox*3 revealed rich genetic diversity only in the populations KC, KS and PK from Thailand (*h*=0.439–0.655; *π*=0.00213–0.00329, **Table 1**). ITS2 showed that all Thai populations exhibited much lower ribotype and nucleotide diversity (*h*=0.000–0.333; *π*=0.00000–0.00061) than the populations SP from Singapore (*h*=0.514; *π*=0.00156) and BL from Indonesia (*h*=0.692; *π*=0.00877) (**Table 1**).

### Population genetic structuring

AMOVA revealed incompatible results among four datasets for *S. plagiophyllum* (**Supplementary Table S2**). *Cox*1 and *cox*1+*cox*3 identified approximate 80–90% of the total genetic variation occurred among populations within groups (*p*<0.001). *Cox*3 revealed 89.19% of the total genetic variation within populations, whereas ITS2 identified 57.99% of the genetic variation among groups, but both were statistically non-significant (**Supplementary Table S2**). For *S. polycystum*, AMOVA indicated that approximate 36–45% of the genetic variation occurred within populations (*p*<0.001), and the rest were distributed among populations within groups (*p*<0.001, **Supplementary Table S2**). *Cox*1+*cox*3 based F_ST_ estimates showed that BK and KH from Koh Lanta Yai, Thailand highly differentiated from other *S. plaiophyllum* populations (**Supplementary Table S3**). For *S. polycystum*, different datasets consistently revealed that SP from Singapore, BL from Indonesia and KB from Thailand significantly diverged from other populations (**Supplementary Table S4**).

**Figure 2:**
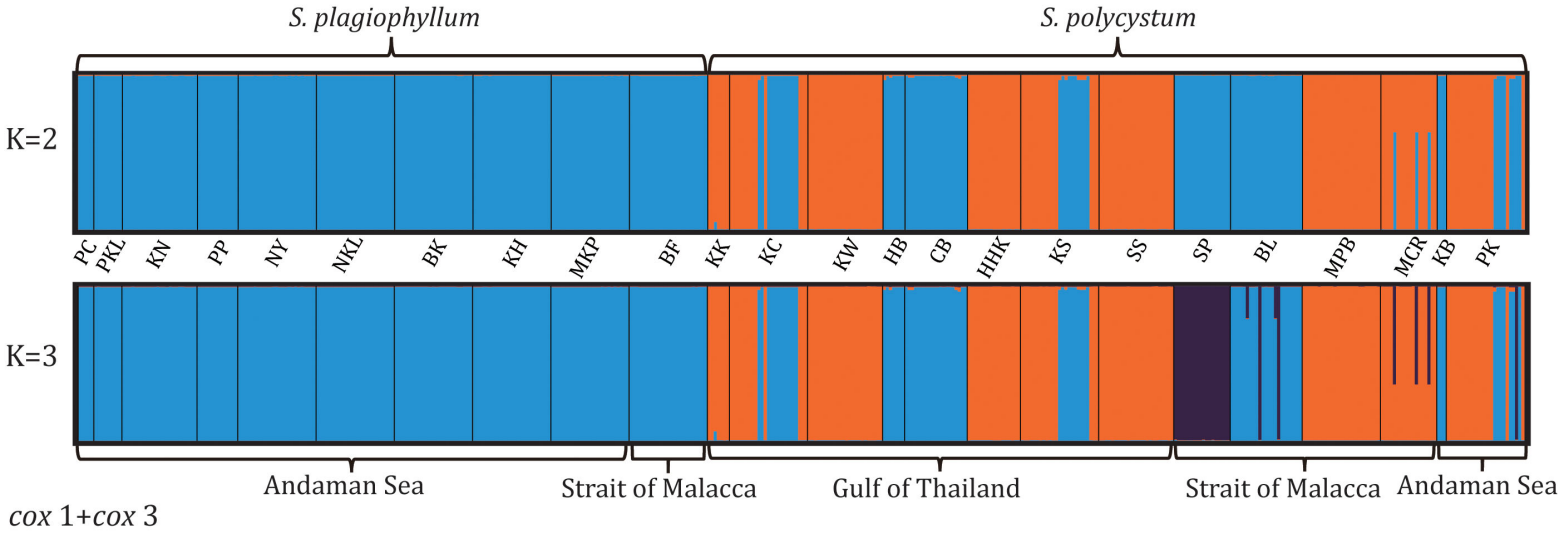
Structure analysis based on mitochondrial *cox*1+*cox*3 of *S. plagiophyllum* and *S. polycystum* at K = 2 **(b)** and K = 3 **(d)**. Population codes are the same as in **Table 1**.

Population structuring analysis of either combined (**Figure 2**) or separated dataset (**Supplementary Figure S3**), revealed the optimal Delta K-value (K=2), suggesting two genetically divided groups (*S. plagiophyllum* vs. *S. polycystum*). *Cox*3/ITS2-based clustering at K=2 indicated amounts of individual admixture between *S. plagiophyllum* and *S. polycystum* (**Supplementary Figure S3B-S3C**), despite *cox*3 showing that all *S. plagiophyllum* populations exclusively clustered as a separate group. Clustering analysis at K=3 revealed subtly structured populations in each *Sargassum* species, particularly for *S. polycystum*. This can mostly be ascribed to the unique genetic characteristics anchored in the populations SP from Singapore and BL from Indonesia (**Figure 2**).

### Historical demography and migration

**Figure 3:**
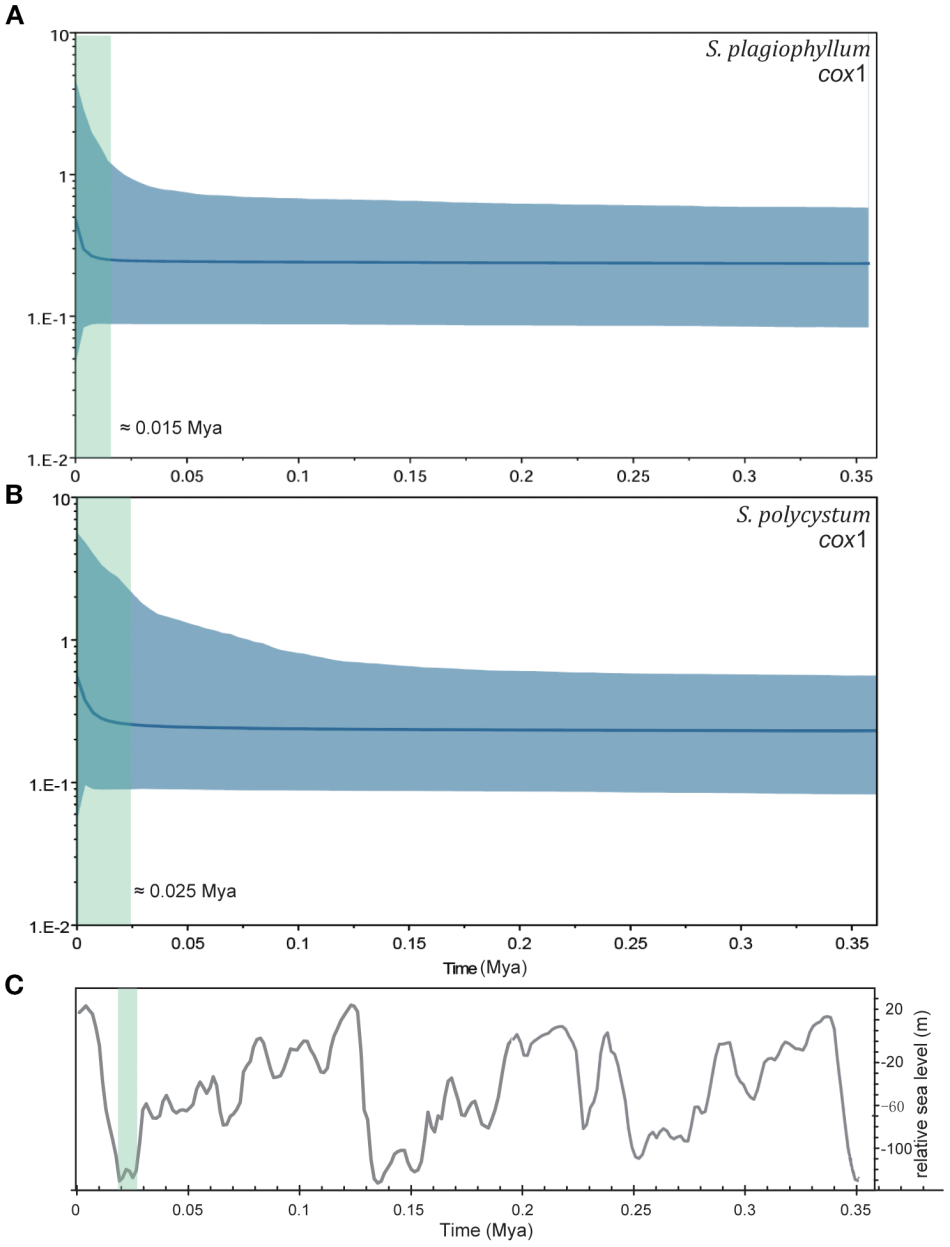
Bayesian skyline plots (BSPs) for *S. plagiophyllum* **(A)** and *S. polycystum* **(B)** based on *cox*1 dataset. Blue lines are the median posterior effective population size through time, blue shaded areas represent 95% confidence intervals. The green shadows represent the approximate demographic expansion time. **(C)** The global relative sea level curve during the late Pleistocene glacial cooling (modified from Waelbroeck et al., 2002).

BSPs results indicated that both *Sargassum* species experienced long-term stabilized population size since 0.35 Mya (**Figure 3**, **Supplementary Figure S4**). *Cox*1-inferred demographic expansion occurred at *c*. 0.015 Mya for *S. plagiophyllum* and at *c*. 0.025 Mya for *S. polycystum* (**Figure 3**). *Cox*3 and ITS2 analysis revealed similar demographic histories, with *S. plagiophyllum* expanded at *c*. 0.015–0.060 Mya (**Supplementary Figure S4A-S4C**) and *S. polycystum* expanded at *c*. 0.025–0.035 Mya (**Supplementary Figure S4B-S4D**).

**Figure 4:**
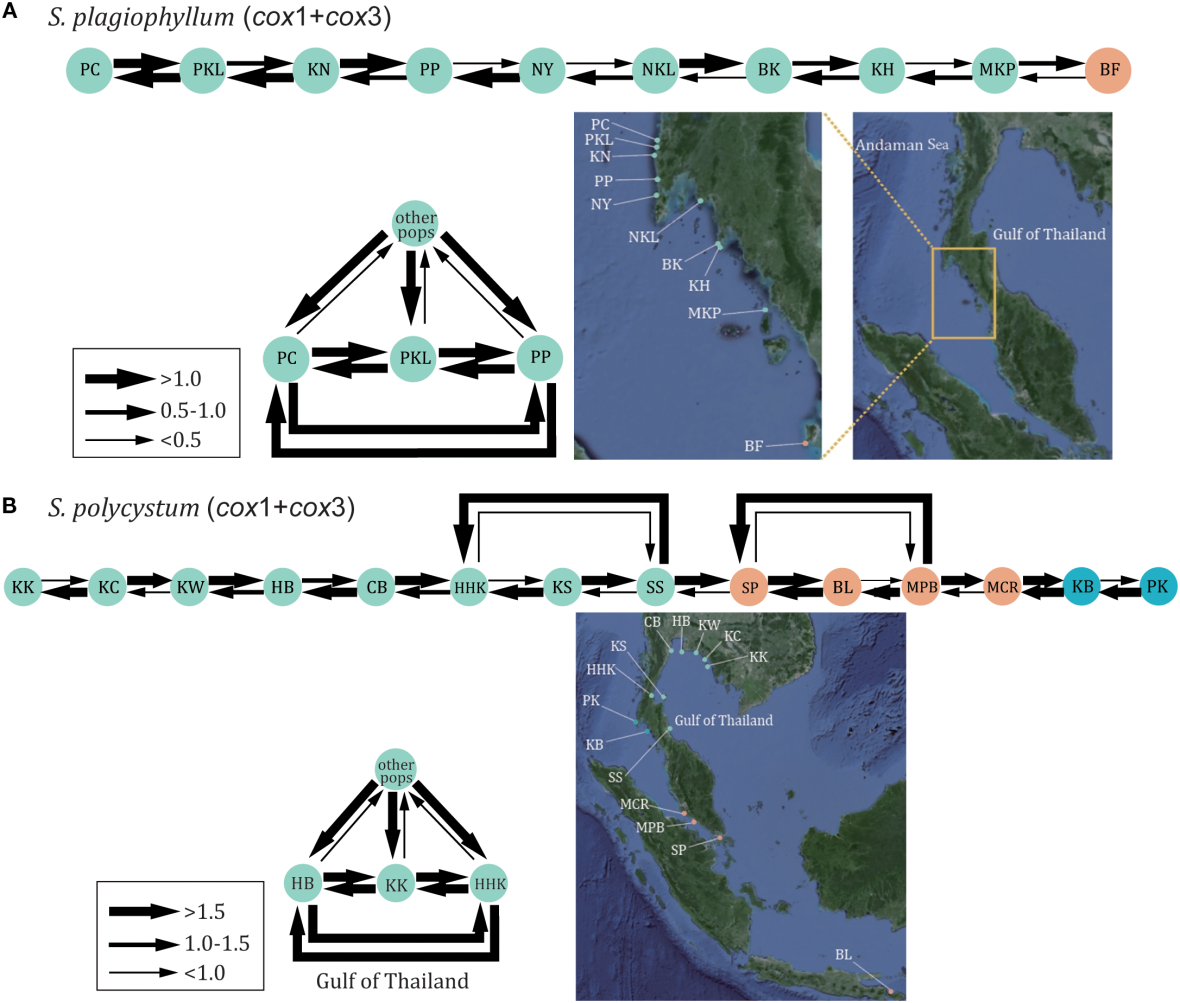
The estimated gene flow between *S. plagiophyllum* populations **(A)** and *S. polycystum* populations **(B)** in the Thai-Malay Peninsula based on *cox*1+*cox*3 dataset.

For *S. plagiophyllum*, mitochondrial and nuclear datasets revealed a primarily south-to-north migration (**Figures 4**, **S5A**, **S6A**, **S7A**). Specifically, there were numerous migrations towards the west coast of Phuket Island (i.e. the PKL population) in the Andaman Sea from the northern Malacca Strait. Instead, the opposite migration from the Phuket Island (e.g. PC, PKL and PP) to other populations were generally weak (**Figures 4A**, **S5A**, **S6A**, **S7A**). For *S. polycystum*, molecular datasets revealed a large amount of clockwise migration between populations in the Gulf of Thailand, despite complementary anticlockwise migration occurred (**Figures 4B**, **S5B**, **S6B**, **S7B**). Intensive migration also occurred among the three populations HB, KK and HHK in the Gulf of Thailand. However, there was no clear migration spanning from Bali Island to the Andaman Sea.

### Network diagrams and molecular dating

**Figure 5:**
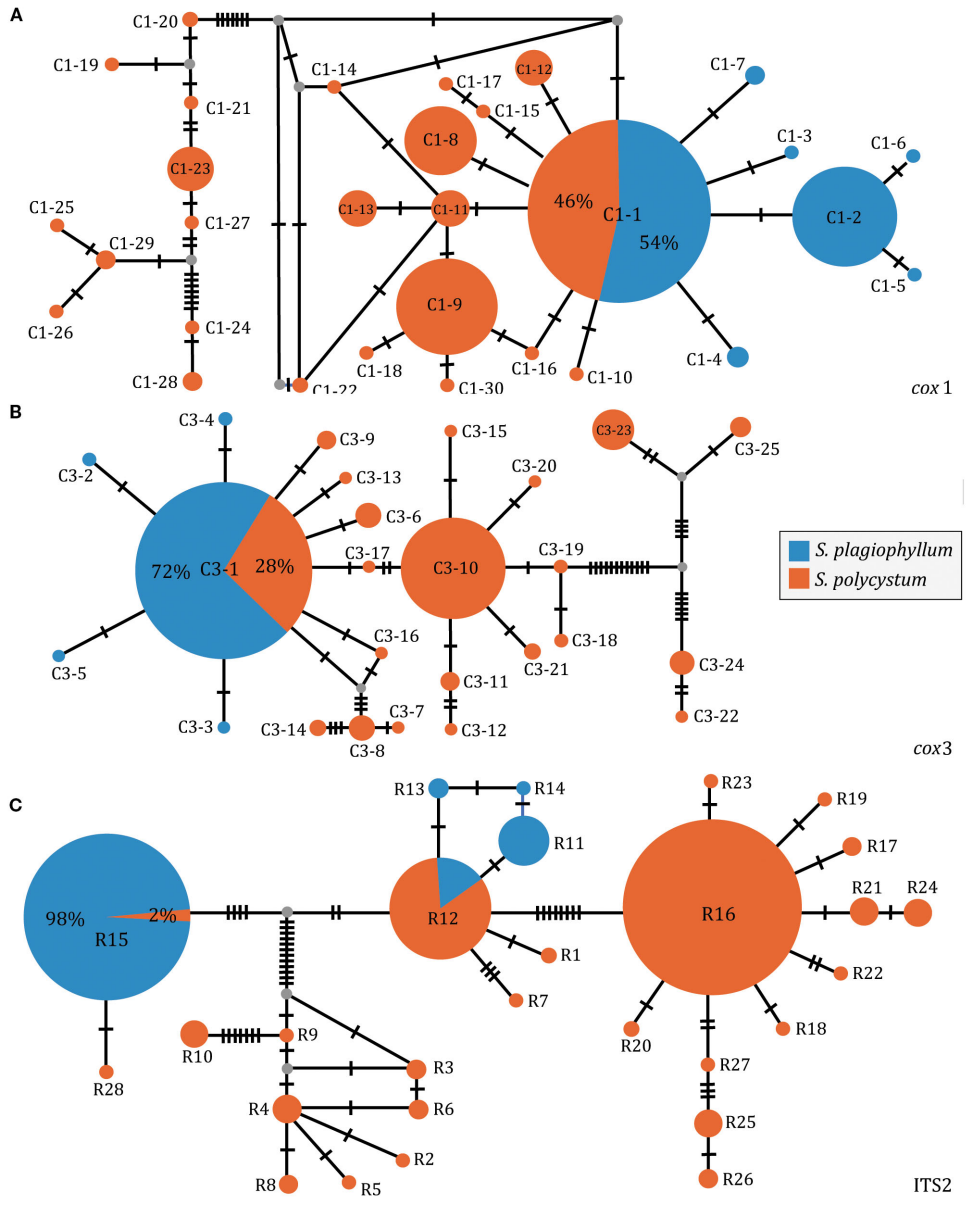
Parsimony median-joining network based on *cox*1 **(A)**, *cox*3 **(B)** and ITS2 **(C)** dataset of *S. plagiophyllum* and *S. polycystum*. Circle size is proportional to population sample size. Each line between haplotypes/ribotypes represents one mutation step.

When pooling each of the three datasets of *S. plagiophyllum* and *S. polycystum* with those from Hainan-Guangxi (China), we identified 30 *cox*1 (C1-1–C1-30, **Figure 5A**) and 25 *cox*3 haplotypes (C3-1–C3-25, **Figure 5B**), and 28 ITS2 ribotypes (R1-R28, **Figure 5C**). The network diagram showed that *cox*1-inferred C1-1, *cox*3-inferred C3-1 as well as ITS2-inferred R12 and R15, were each shared between *S. plagiophyllum* and *S. polycystum* in distribution (**Figure 5**), which accounted for 52.81% (442/837), 54.30% (467/860), and 26.32% (215/817) of the total individuals analyzed, respectively. *Cox*1, *cox*3 and ITS2 consistently showed that some haplotypes/ribotypes from Hainan-Guangxi (China) formed an independent structured lineage that was evidently diverged from other haplotypes/ribotypes (**Supplementary Figure S8**). The three datasets also indicated that the most common distributed *cox*1/*cox*3-inferred C1-1/C3-1 and ITS2-inferred R16 were co-occurred widely around the TMP populations (the Andaman Sea, the Gulf of Thailand and the Malacca Strait, **Supplementary Figure S8**).

Molecular dating showed that the *S. plagiophyllum*-*S. polycystum* clade diverged *c*. 0.43–0.49 Mya (**Supplementary Figures S9**, **S10**, **S11**), which is compatible with the estimation by Yip et al. (2020). Building on this dating insight, the genetically isolated lineage of *S. polycystum* from Hainan-Guangxi (China) was estimated to occur and diversify *c*. 0.26–0.31 Mya. Over the TMP, *S. polycystum* haplotypes/ribotypes are nested within *S. plagiophyllum* stochastically, and started to diverge as a separate lineage *c.* 0.17–0.23 Mya (**Supplementary Figures S9**, **S10**, **S11**).

## Discussion

### The late Pleistocene glacial survival and post-glacial expansion

Phylogeographic evidence (**Figures 1**, **3**, **S2**, **S4**, **S9**-**S11**) showed that the origination and demographic expansion of *S. polycystum*/*S. plagiophyllum* around the TMP were likely linked to sea level fluctuations driven by the late Pleistocene glacial-interglacial cycles. The highest numbers of endemic haplotypes/ribotypes and highest genetic diversity in PKL, KN and NY (**Table 1**, **Figures 1**, **S2**) suggest that the Andaman Sea (e.g. the west coast of the Phuket Island) probably harboured a glacial refugium for the ancestral population of *S. plagiophyllum* to survive (Provan et al., 2005; Maggs et al., 2008; Hu et al., 2018), when the maximum sea levels dropped to -120 meters in the Sunda shelf during the maximum glaciation (Voris, 2000; Woodruff, 2010).

For *S. polycystum*, two potential glacial refugia have been proposed for its ancestral survival during the late Pleistocene sea-level fluctuations (Hu et al., 2018), with one in the southwest of Hainan Island, China and the other distributed around the western Coral Triangle (e.g. the Bali Island, Indonesia). Here, the combined network diagrams (**Supplementary Figure S8**) and molecular dating of *S. plagiophyllum*/*S. polycystum* (**Supplementary Figure S9**; **Supplementary Figure S11**) revealed an evolutionary distinctiveness of Hainan-Guangxi haplotypes/ribotypes, providing additional evidence of a refugium existed near Hainan Island. Moreover, *cox*1-based *S. polycystum* haplotypes (C1-6–C1-11) were found only in the populations MCR, MPB and SP in the Malacca Strait (**Figure 1D**). Such a uniqueness of genetic diversity, together with the exclusive occurrence of haplotypes in the population BF for *S. plagiophyllum* (i.e. C1-4 in **Figure 1A**, C3-4 in **Figure 1C**, and H11 in **Supplementary Figure S2A**), suggest the likely existence of a third glacial refugium for *S. polycystum* in the northern Malacca Strait (Voris, 2000; Cannon et al., 2009; Wee et al., 2020). Alternatively, the rich genetic variation in the Malacca Strait probably arose from the secondary contact of other divergent lineages of *S. polycystum* as reported in the mangrove *Sonneratia alba* Sm (Yang et al., 2017). during inter-glacial periods. However, discerning these biogeographic scenarios depends on the integration of more evidence of paleogeographic coastal configuration during the ice ages, including more samplings of periphery populations like south Java, Sunda Strait, West Sumatra, Myanmar and Coral Triangle, together with phylogeographic illustrations from other species co-occurred around the TMP.

The *S. polycystum* and *S. plagiophyllum* clade was estimated to originate from the common ancestor at *c*. 0.43–0.49 Mya. Afterwards, the lineage of *S. polycystum* in Hainan-Guangxi (China) formed *c*. 0.26–0.31 Mya, and another lineage of combined *S. polycystum* and *S. plagiophyllum* around the TMP occurred *c*. 0.17–0.23 Mya (**Supplementary Figures S9**–**S11**). These timeframes correspond to the late Pleistocene epoch and are compatible to the latest organellar-genome based interspecific time-calibration (0.10–0.53 Mya) (Zhang et al., 2022), including the historical biogeographic dating of the most recent diverged section *Polycystae* at *c*. 0.40 Mya (**Figure 1** in Yip et al., 2020). In addition, both *Sargassum* species around the TMP experienced slight demographic expansion *c*. 0.012–0.055 Mya (**Figures 3**, **S4**), which corresponds to the transition from the last glaciation (*c*. 0.115–0.012 Mya) in the late Pleistocene to early Holocene. It should be noted that the demographic expansion spectrums of *S. polycystum* around the TMP are much later than previous mismatch-distribution based estimation (0.41–0.62 Mya, Chan et al., 2013) and the *cox*1+*cox*3 based extend-BSPs results in Hainan-Guangxi (China) (0.60–0.80 Mya, Hu et al., 2018). It is also later than the expansion time in the congeneric *S. aquifolium* in Southeast Asia (0.25 Mya, Chan et al., 2014).

### Ocean currents and population genetic connectivity

The most common haplotype/ribotype of *S. polycystum* (C1-1 in **Figure 1D**, R16 in **Figure 1E**, C3-10 in **Figure 1F**, H14 in **Supplementary Figure S2B**) distributes widely on both sides of the TMP. The lack of east-west differentiation indicates that the TMP has not served as a contemporary geographical barrier affecting genetic connectivity of *S. polycystum*. This contrasts with recent studies that illustrated a distinct genetic break across the TMP in mangroves with lower dispersal capabilities (e.g., *Avicennia alba* Blume and *Sonneratia alba*, Yang et al., 2017; Wee et al., 2020). However, our finding is consistent with the reported absence of an east-west genetic differentiation in the mangrove *Rhizophora mucronata* Lam. with high dispersal potential via progagules (Wee et al., 2014). The observed phylogeographic diversity and genetic structure do not present evidence of vicariance in *S. polycystum*/*S. plagiophyllum* (Wee et al., 2014; Tan et al., 2018). Instead, the widespread distribution of the ancestral haplotype/ribotype in the west of the TMP for *S. plagiophyllum* (C1-1 in **Figure 1A**, R15 in **Figure 1B**, C3-1 in **Figure 1C**, H4 in **Supplementary Figure S2A**), together with the structuring analysis (**Supplementary Figure S3**) and gene flow estimation (**Figures 4**, **S5**-**S7**), further allow us to propose that post-glacial genetic exchange driven by oceanic current may have re-established population connectivity in each *Sargassum* species across the TMP.

Regardless of migration pathway and recent migration rate, both *Sargassum* species exhibited a stepping-stone migration pattern along the Malacca Strait (**Figures 4**, **S5**-**S7**), which is basically in accordance with the correlation between geographical and genetic distance along a linear coastline observed in mangroves (Kennedy et al., 2017; Wee et al., 2020). The clear south-to-north historical migration route observed in *S. plagiophyllum*/*S. polycystum* along the Malacca Strait (**Figures 4**, S5-S7) can be explained by a strong contemporary gene flow via sea dispersal, as it concurs with the present-day ocean circulation patterns that predominantly flow from south to north in the Malacca Strait (Rizal et al., 2012). In the Gulf of Thailand, a high proportion of recent migration between distant *S. polycystum* populations indicates continuous contemporary gene flow driven by the clockwise circulation during the northeast monsoon season and a counterclockwise vortex during the southwest monsoon.

*Sargassum* species (e.g. *S. plagiophyllum*) around the TMP has a cycle of growth and reproduction throughout the year (Pongparadon et al., 2019) and reached its peak percentage of fertility at the end of the north-east monsoon season (Prathep et al., 2019). As the late Pleistocene ice age gradually ended, sea-level rose and connectivity between the east and west sides of the TMP was restored (Hall, 2009). The rise in temperature during the inter-glacial periods facilitated more favorable conditions enabling the detached vegetative branches of *S. polycystum*/*S. plagiophyllum* to float, survive and migrate (Komatsu et al., 2007; Woodruff, 2010). Particularly during the north-east and south-west monsoons, strong currents flow from the South China Sea via the Malacca Strait to the Indian Ocean (**Figure 1**). Thus, the long-distance dispersal can be considerably accelerated by seasonal monsoons that augment the ocean currents (Komatsu et al., 2007). These current systems not only prevented the mixing of waters at the boundary between the south of the Andaman Sea and the north of the Malacca Strait (Wee et al., 2014), but also likely contributed to *S. polycystum* and S. *plagiophyllum* to expand their distribution ranges north up to the Myanmar Andaman Sea and on the Gulf of Bengal coast (Soe-Htun et al., 2012).

### Genetic signatures of incipient speciation

Repeated ecologically-driven transitions during the late Pleistocene glaciations are recognized as an important factor to generate genetic divergence among seaweed populations, ultimately leading to multiple speciation events via repeated isolation in allopatric refuges (Fraser et al., 2016; Ayres-Ostrock et al., 2019; Neiva et al., 2024). In the case of the sister-species *S. polycystum* and *S. plagiophyllum*, they occur largely allopatric around the TMP. *Sargassum plagiophyllum* occurs in abundance in the intertidal along the entire west coast of Thailand and is also recorded from the Andaman coast of Myanmar. Its southern limit is Penang Island (Malaysia) in the northern Malacca Strait. *Sargassum polycystum* occurs in the shallow subtidal of the Gulf of Thailand and in the intertidal in the southern Malacca Strait. It was not found in Lanta Island, where it was collected in the past (Kantachumpoo et al., 2015), which is likely linked to human-mediated change of the maximum sea surface salinity, dissolved oxygen and phosphate concentration (Sumandiarsa et al., 2021; Stankovic et al., 2022). The small intertidal *S. polycystum* population Phuket (PK) grew mixed with *S. ilicifolium*, close to the low water edge and would only be exposed during springtide. At the same site, but a little bit higher grew *S. plagiophyllum* (NY) in abundance. *Sargassum polycystum* is not found along the Andaman coast of Myanmar. *Sargassum plagiophyllum* grows all year round and reproduces during November-February (Pongparadon et al., 2019), whereas *S. polycystum* can only be found during the short reproductive season in July in Phuket (Thongroy et al., 2007). These ecological-niche and phenological transitions may be a directional selection process that was initiated by divergence in allopatric conditions during glacial phases (Fraser et al., 2016; Cocito et al., 2019), presenting important signatures of local adaptation and reproductive isolation in incipient species (Futuyma, 2005; Charron et al., 2014).

At present, the low mitochondrial resolution prevents confirmation of the degree of reproductive isolation of *S. polycystum*/*S. plagiophyllum* and, therefore, the underlying evolutionary scenarios behind their incipient speciation remain unclear. However, vicariance events, for example the repeated sea-level fluctuations in the Southeast Asia during the late Pleistocene (Benzie, 1998; Woodruff, 2010) followed by ‘founder takes all’ density-dependent processes during post-glacial range expansion (Waters et al., 2013) that can act as a plausible explanation to niche and reproductive season differentiation. In addition, parallel divergence underpinned by historical peripheral isolation and niche differentiation (e.g. temperature, Isa et al., 2020) seems a key force in driving allopatric speciation and radiation of hermaphrodite lineages across the TMP (Almeida et al., 2022), particularly for the dioecious *S. plagiophyllum* and *S. polycystum* with peripheral isolation of range edge and directional introgressive gene flow (BF in **Figures 1C** and S2A, **Supplementary Table S3**). The wide sharing of the common haplotypes/ribotypes between *S. polycystum* and *S. plagiophyllum* suggests that incipient reproductive isolation may have evolved as a by-product of divergence in allopatric speciation (Futuyma, 2005), representing an additional example of incipient reproductive speciation in the brown algae of the order Fucales (Neiva et al., 2024). In the future, genome-scale screening of independent replicating SNPs can be considered to identify clear evidence of recent or incipient speciation (Almeida et al., 2022).

## Conclusion

Series of phylogeographic evidence illustrated that the biogeographic and genetic variation patterns of *S. plagiophyllum*/*S. polycystum* around the TMP likely resulted from ancestral survival during the late Pleistocene ice ages and following circulation-driven demographic expansion. Contemporary gene flow and population genetic structuring are maintained by ocean-current facilitating long-distance dispersal of detached rafting thalli. The prevailing ocean currents in the Gulf of Thailand and the Malacca Strait ostensibly explain the directions of population-level genetic continuity. Mitochondrial and nuclear datasets exhibited signature of incipient differentiation between *S. plagiophyllum* and *S. polycystum*. Future wider-sampling based phylogeographic studies of seaweeds are important and imperative, by incorporating oceanographic modelling approaches (Wee et al., 2014; Liang et al., 2022), to empirically confirm the influence of dispersal potential on the long-term patterns of population connectivity.

## Data Availability

The datasets presented in this study can be found in online repositories. The names of the repository/repositories and accession number(s) can be found in the article/**Supplementary Material**.
