# Supplementary material for: Comparative phylogeographic patterns and processes of the incipient brown seaweeds Sargassum polycystum and S. plagiophyllum around the Thai-Malay Peninsula
Source: Front Plant Sci. 2025 Sep 17;16:1673650. doi: 10.3389/fpls.2025.1673650 (PMC12484222; doi:10.3389/fpls.2025.1673650)
Supplement: Supplementary file 1 [file DataSheet1.docx]

**Comparative phylogeographic patterns and processes of the incipient brown seaweeds *Sargassum polycystum* and *S. plagiophyllum* around the Thai-Malay Peninsula**

Supplementary materials

**I. TABLES**

**TABLE S1** Primer sets used to amplify mitochondrial and nuclear markers in this study.

| Markers | Primers | Sequences | Source |
| --- | --- | --- | --- |
| *cox*1 | GazF2 | 5’-CAACCAYAAAGATATWGGTAC-3’ | Lane *et al.*, 2007 |
|  | GazR2 | 5’-GGATGACCAAARAACCAAAA-3’ | Lane *et al.*, 2007 |
| *cox*3 | trnY-P1 | 5’-TCYATCRTAGGTTCGAATCC-3’ | Kogishi *et al.*, 2010 |
|  | Cox3-P2 | 5’-ACAAARTGCCAATACCAAGC-3’ | Kogishi *et al.*, 2010 |
| ITS2 | 5.8S-BF | 5’-CGATGAAGAACGCAGCGAAATGCGAT-3’ | Yoshida *et al.*, 2000 |
|  | 25BR-2 | 5’-TCCTCCGCTTAGTATATGCTTAA-3’ | Yoshida *et al.*, 2000 |

W= A or T, R= A or G, Y= C or T

**TABLE S2** Analysis of molecular variance (AMOVA) to partition genetic variance in *Sargassum polycystum* and *S. plagiophyllum* based on *cox*1, *cox*3 and ITS2.

| *Sargassum plagiophyllum* | Among groups | | | Among populations within groups | | | Within populations | | |
| --- | --- | --- | --- | --- | --- | --- | --- | --- | --- |
|  | d.f. | Var(%) | ɸ_CT_ | d.f. | Var(%) | ɸ_SC_ | d.f. | Var(%) | ɸ_ST_ |
| *cox*1 | 1 | -25.81 | -0.25806 | 8 | 89.19 | 0.70893^***^ | 329 | 36.62 | 0.63381^***^ |
| *cox*3 | 1 | 9.55 | 0.09546 | 8 | 1.26 | 0.01398^*^ | 331 | 89.19 | 0.10811 |
| *cox*1+*cox*3 | 1 | -22.91 | -0.22914 | 8 | 81.87 | 0.66608^***^ | 329 | 41.04 | 0.58957^***^ |
| ITS2 | 1 | 57.99 | 0.57991 | 7 | 30.67 | 0.73005^***^ | 277 | 11.34 | 0.88660^***^ |
| *Sargassum polycystum* | Among groups | | | Among populations within groups | | | Within populations | | |
|  | d.f. | Var(%) | ɸ_CT_ | d.f. | Var(%) | ɸ_SC_ | d.f. | Var(%) | ɸ_ST_ |
| *cox*1 | 2 | 12.90 | 0.12896 | 11 | 42.14 | 0.48384^***^ | 312 | 44.96 | 0.55041^***^ |
| *cox*3 | 2 | -17.27 | -0.17271 | 11 | 79.77 | 0.68022^***^ | 322 | 37.50 | 0.62500^***^ |
| *cox*1+*cox*3 | 2 | -7.74 | -0.07737 | 11 | 68.56 | 0.63639^***^ | 296 | 39.17 | 0.60826^***^ |
| ITS2 | 2 | -12.49 | -0.12485 | 11 | 76.36 | 0.67881^***^ | 327 | 36.13 | 0.63871^***^ |

d.f.: degree of freedom, Var(%): percentage of variation, ^***^: *P* < 0.001; ^**^: *P* < 0.01; ^*^: *P* < 0.05

**TABLE S3** Population pairwise F_ST_ estimation based on *cox*1+*cox*3 (upper) and ITS2 (lower) for *S. plagiophyllum*. Highly differentiated values are highlighted in grey shaded in background.

|  |  | PC | PKL | KN | PP | NY | NKL | BK | KH | MKP | BF |
| --- | --- | --- | --- | --- | --- | --- | --- | --- | --- | --- | --- |
| *cox*1+*cox*3 | PC | 0.0000 |  |  |  |  |  |  |  |  |  |
|  | PKL | -0.0778 | 0.0000 |  |  |  |  |  |  |  |  |
|  | KN | 0.0334 | 0.0978 | 0.0000 |  |  |  |  |  |  |  |
|  | PP | 0.0000 | 0.0429 | 0.1244 | 0.0000 |  |  |  |  |  |  |
|  | NY | -0.0518 | 0.0192 | 0.0478 | 0.0232 | 0.0000 |  |  |  |  |  |
|  | NKL | 0.0000 | 0.1816 | 0.2266^***^ | 0.0000 | 0.0735 | 0.0000 |  |  |  |  |
|  | BK | 0.7606^***^ | 0.7511^***^ | 0.4443^***^ | 0.8057^***^ | 0.6944^***^ | 0.8697^***^ | 0.0000 |  |  |  |
|  | KH | 0.8768^***^ | 0.8623^***^ | 0.5864^***^ | 0.8902^***^ | 0.7789^***^ | 0.9145^***^ | -0.0113 | 0.0000 |  |  |
|  | MKP | -0.1009 | 0.0797 | 0.2903^***^ | -0.0299 | 0.0791 | -0.0019 | 0.8685^***^ | 0.8957^***^ | 0.0000 |  |
|  | BF | -0.0301 | 0.0457 | 0.1639^***^ | 0.0508 | 0.1045^***^ | 0.1197 | 0.7085^***^ | 0.8007^***^ | 0.1577^***^ | 0.0000 |
|  |  | PC | PKL | KN | PP | NY | BK | KH | MKP | BF |  |
| ITS2 | PC | 0.0000 |  |  |  |  |  |  |  |  |  |
|  | PKL | 0.6486 | 0.0000 |  |  |  |  |  |  |  |  |
|  | KN | 0.0492 | 0.4446 | 0.0000 |  |  |  |  |  |  |  |
|  | PP | 0.9333^***^ | 0.1125 | 0.7179^***^ | 0.0000 |  |  |  |  |  |  |
|  | NY | 0.5589^***^ | -0.0444 | 0.4118^***^ | 0.0817 | 0.0000 |  |  |  |  |  |
|  | BK | 0.0000 | 0.8304^***^ | 0.1939 | 0.9677^***^ | 0.6660^***^ | 0.0000 |  |  |  |  |
|  | KH | 0.0000 | 0.9134^***^ | 0.3295 | 0.9836^***^ | 0.7653^***^ | 0.0000 | 0.0000 |  |  |  |
|  | MKP | 0.0000 | 0.9445^***^ | 0.4346 | 0.9895^***^ | 0.8247^***^ | 0.0000 | 0.0000 | 0.0000 |  |  |
|  | BF | 0.9398^***^ | 0.2251 | 0.7935^***^ | 0.0685 | 0.1954^***^ | 0.9604^***^ | 0.9759^***^ | 0.9833^***^ | 0.0000 |  |

^***^：*P* < 0.001

**TABLE S4** Population pairwise F_ST_ estimation based on *cox*1+*cox*3 (upper) and ITS2 (lower) for *S. polycystum*. Highly differentiated values are highlighted in grey shaded in background.

|  |  | KK | KC | KW | HB | CB | HHK | KS | SS | SP | BL | MPB | MCR | KB | PK |
| --- | --- | --- | --- | --- | --- | --- | --- | --- | --- | --- | --- | --- | --- | --- | --- |
| *cox*1+*cox*3 | KK | 0.0000 |  |  |  |  |  |  |  |  |  |  |  |  |  |
|  | KC | 0.2389 | 0.0000 |  |  |  |  |  |  |  |  |  |  |  |  |
|  | KW | 0.0394 | 0.3382^***^ | 0.0000 |  |  |  |  |  |  |  |  |  |  |  |
|  | HB | 0.7526^***^ | 0.4022 | 0.8667^***^ | 0.0000 |  |  |  |  |  |  |  |  |  |  |
|  | CB | 0.7702^***^ | 0.4566^***^ | 0.8435^***^ | 0.0453^***^ | 0.0000 |  |  |  |  |  |  |  |  |  |
|  | HHK | 0.0319 | 0.3166^***^ | -0.0477 | 0.8512^***^ | 0.8302^***^ | 0.0000 |  |  |  |  |  |  |  |  |
|  | KS | 0.1271 | 0.0858 | 0.1510 | 0.4930^***^ | 0.5532^***^ | 0.1244 | 0.0000 |  |  |  |  |  |  |  |
|  | SS | 0.0660 | 0.3492^***^ | -0.0261 | 0.8543^***^ | 0.8374^***^ | -0.0492 | 0.1366 | 0.0000 |  |  |  |  |  |  |
|  | SP | 0.9212^***^ | 0.8371^***^ | 0.9479^***^ | 0.8816^***^ | 0.8714^***^ | 0.9436^***^ | 0.8340^***^ | 0.9441^***^ | 0.0000 |  |  |  |  |  |
|  | BL | 0.6823^***^ | 0.4644^***^ | 0.7716^***^ | 0.1651^***^ | 0.2361^***^ | 0.7487^***^ | 0.5578^***^ | 0.7686^***^ | 0.7804^***^ | 0.0000 |  |  |  |  |
|  | MPB | 0.6912^***^ | 0.6657^***^ | 0.7882^***^ | 0.8968^***^ | 0.8775^***^ | 0.7815^***^ | 0.6084^***^ | 0.7811^***^ | 0.9530^***^ | 0.8341^***^ | 0.0000 |  |  |  |
|  | MCR | 0.1849 | 0.4074^***^ | 0.3993^***^ | 0.6683^***^ | 0.7143^***^ | 0.3658^***^ | 0.3772^***^ | 0.4062^***^ | 0.8436^***^ | 0.6858^***^ | 0.3441^***^ | 0.0000 |  |  |
|  | KB | 0.7784 | 0.3400 | 0.8969^***^ | -0.1694 | -0.0940 | 0.8894^***^ | 0.4671 | 0.8816^***^ | 0.8993^***^ | 0.0521 | 0.9180^***^ | 0.6498 | 0.0000 |  |
|  | PK | 0.2148 | -0.0140 | 0.2997^***^ | 0.2805 | 0.3334^***^ | 0.2834^***^ | 0.1157 | 0.3121^***^ | 0.7693^***^ | 0.3875^***^1 | 0.5648^***^ | 0.3583^***^ | 0.2014 | 0.0000 |
|  |  | KK | KC | KW | HB | CB | HHK | KS | SS | SP | BL | MPB | MCR | KB | PK |
| ITS2 | KK | 0.0000 |  |  |  |  |  |  |  |  |  |  |  |  |  |
|  | KC | -0.0422 | 0.0000 |  |  |  |  |  |  |  |  |  |  |  |  |
|  | KW | 0.0000 | -0.0084 | 0.0000 |  |  |  |  |  |  |  |  |  |  |  |
|  | HB | -0.0278 | -0.0335 | 0.0210 | 0.0000 |  |  |  |  |  |  |  |  |  |  |
|  | CB | -0.0334 | 0.0062 | 0.0093 | 0.0006 | 0.0000 |  |  |  |  |  |  |  |  |  |
|  | HHK | 0.0000 | -0.0170 | 0.0000 | 0.0067 | -0.0026 | 0.0000 |  |  |  |  |  |  |  |  |
|  | KS | -0.0353 | 0.0097 | -0.0039 | 0.0080 | 0.0161 | -0.0117 | 0.0000 |  |  |  |  |  |  |  |
|  | SS | 0.0000 | -0.0084 | 0.0000 | 0.0210 | 0.0093 | 0.0000 | -0.0039 | 0.0000 |  |  |  |  |  |  |
|  | SP | 0.8011^***^ | 0.8270^***^ | 0.8536^***^ | 0.8056^***^ | 0.7950^***^ | 0.8371^***^ | 0.8472^***^ | 0.8536^***^ | 0.0000 |  |  |  |  |  |
|  | BL | 0.4630^***^ | 0.5692^***^ | 0.5518^***^ | 0.5056^***^ | 0.5007^***^ | 0.5223^***^ | 0.6266^***^ | 0.5518^***^ | 0.6644^***^ | 0.0000 |  |  |  |  |
|  | MPB | 0.0000 | -0.0007 | 0.0000 | 0.0354 | 0.0210 | 0.0000 | 0.0028 | 0.0000 | 0.8693^***^ | 0.5823^***^ | 0.0000 |  |  |  |
|  | MCR | 0.0899 | 0.1249 | 0.1641 | 0.1084 | 0.1028 | 0.1400 | 0.1451 | 0.1641 | 0.7099^***^ | 0.5280^***^ | 0.1897 | 0.0000 |  |  |
|  | KB | 0.9821^***^ | 0.9729^***^ | 0.9903^***^ | 0.9726^***^ | 0.9611^***^ | 0.9882^***^ | 0.9751^***^ | 0.9903^***^ | 0.9461^***^ | 0.1497 | 0.9920^***^ | 0.9493^***^ | 0.0000 |  |
|  | PK | -0.0463 | 0.0024 | -0.0173 | 0.0009 | 0.0111 | -0.0241 | 0.0099 | -0.0173 | 0.8544^***^ | 0.6425^***^ | -0.0118 | 0.1487 | 0.9765*** | 0.0000 |

^***^：*P* < 0.001

**II. FIGURES**

**
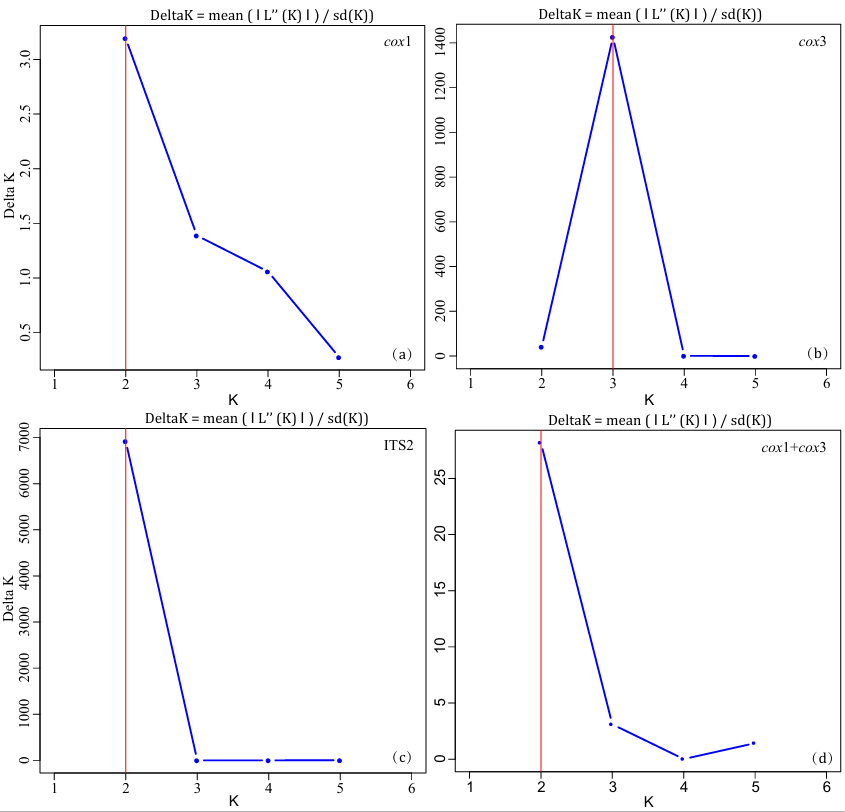
**

**FIGURE S1** The rate of change in ΔK from 1 to 5 for the concatenated *cox*1 (a), *cox*3 (b), ITS2 (c) and *cox*1+*cox*3 (d) sequences of *S. plagiophyllum* and *S. polycystum*, as calculated using STRUCTURE clustering.

**
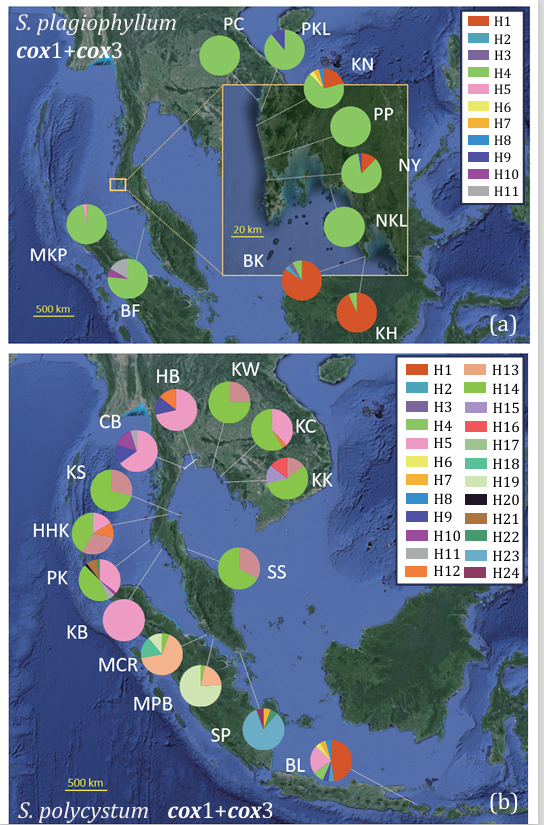
**

**FIGURE S2** Geographical distribution and relative frequency of *cox*1+*cox*3 haplotypes for *S. plagiophyllum* (a) and *S. polycystum* (b).


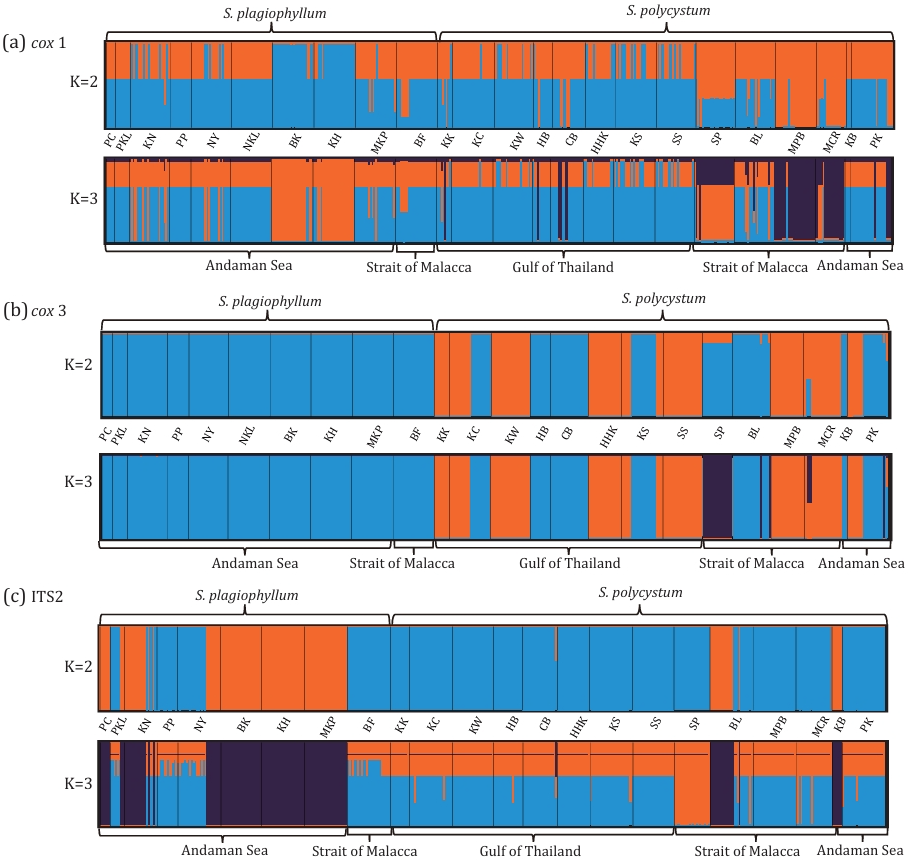


**FIGURE S3** STRUCTURE profiling based on *cox*1 (a), *cox*3 (b) and ITS2 (c) datasets of *S. plagiophyllum* and *S. polycystum* at K = 2 and K = 3. Population codes are the same as in Table 1.


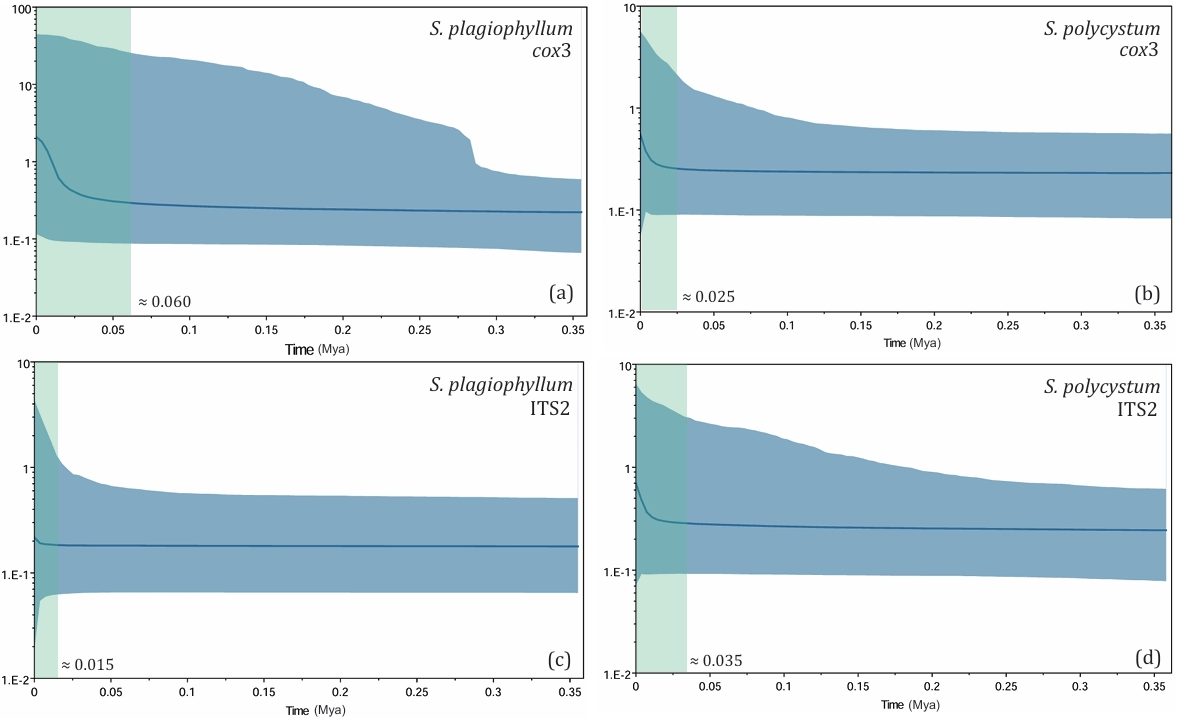


**FIGURE S4** Bayesian skyline plots (BSPs) for *S. plagiophyllum* (a, c) and *S. polycystum* (b, d) from the Thai-Malay Peninsula based on *cox*3 and ITS2 datasets. Blue lines are the median posterior effective population size through time, blue shaded areas represent 95% confidence intervals. The green shadows represent the approximate demographic expansion time.


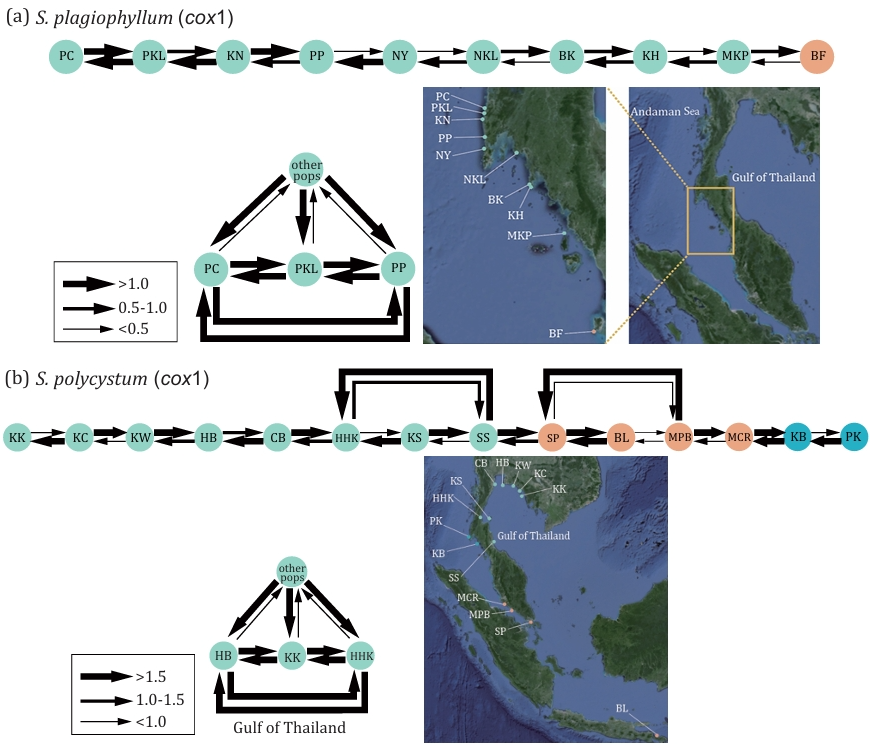


**FIGURE S5** The estimated gene flow between *S. plagiophyllum* populations (a) and *S. polycystum* populations (b) around the Thai-Malay Peninsula based on *cox*1 dataset.


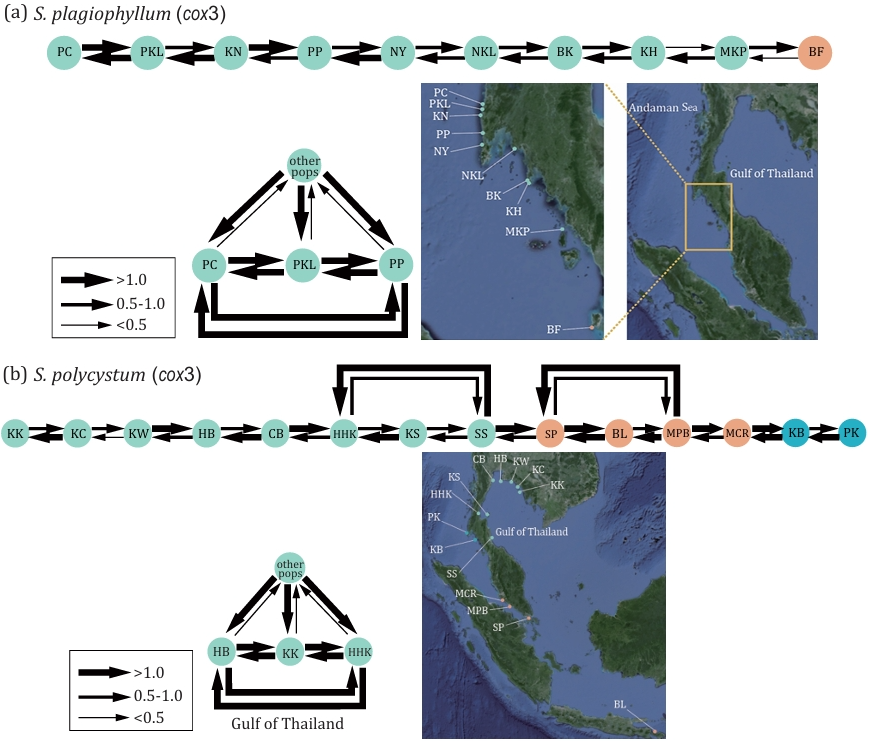


**FIGURE S6** The estimated gene flow between *S. plagiophyllum* populations (a) and *S. polycystum* populations (b) around the Thai-Malay Peninsula based on *cox*3 dataset.


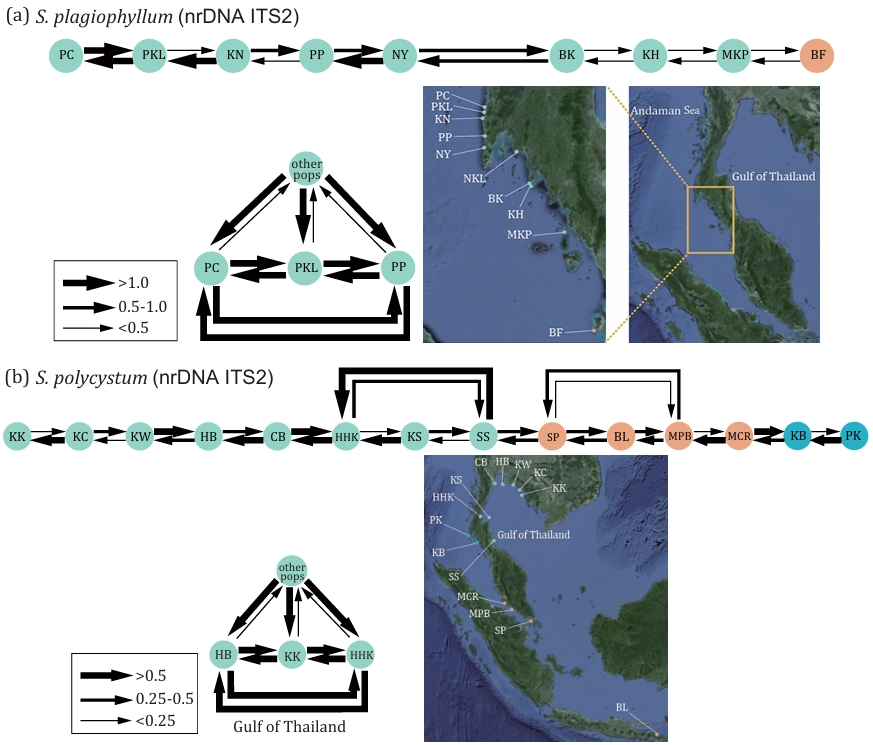


**FIGURE S7** The estimated gene flow between *S. plagiophyllum* populations (a) and *S. polycystum* populations (b) around the Thai-Malay Peninsula based on ITS2 dataset.


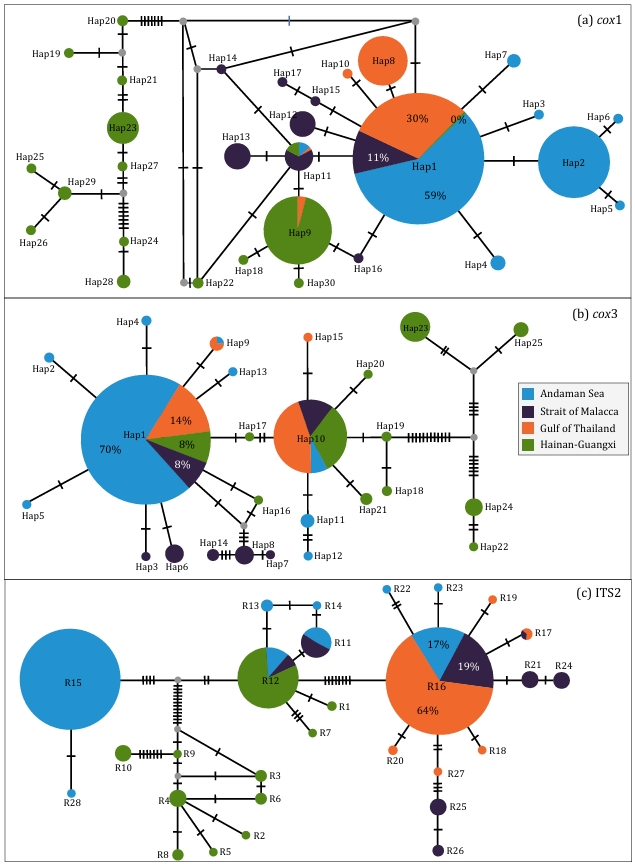


**FIGURE S8** Parsimony median-joining network based on *cox*1 (a), *cox*3 (b) and ITS2 (c) dataset for *S. plagiophyllum* and *S. polycystum*. Circle size is proportional to population sample size. Each line between haplotypes represents one mutation step.


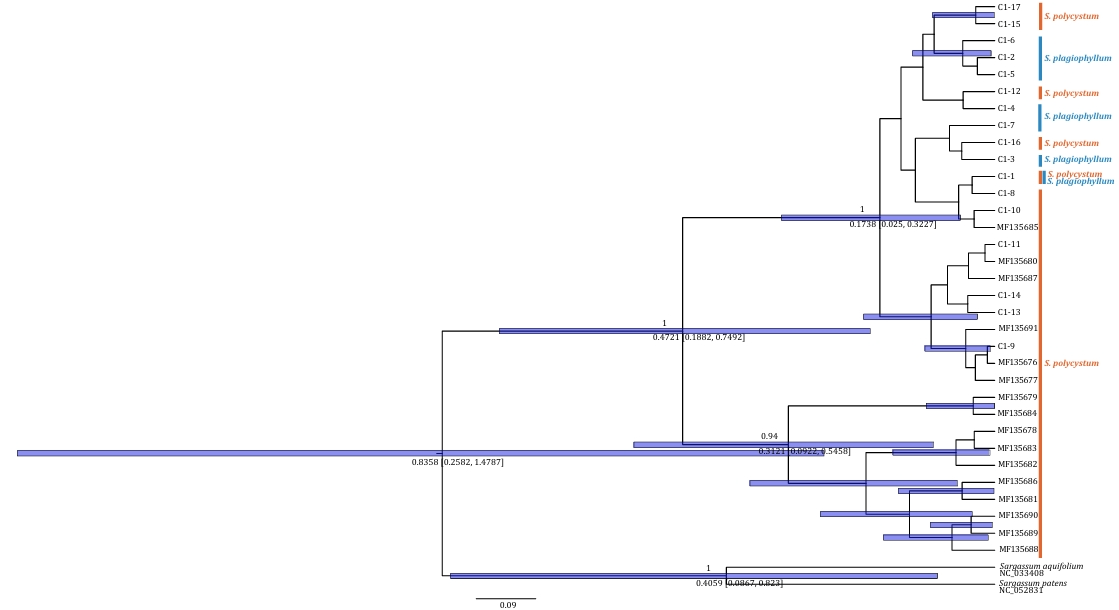


**FIGURE S9** Divergence time estimated among *cox*1 haplotypes of *S. plagiophyllum* and *S. polycystum*. The blue bars represent 95% confidence interval. Unique genotypes showed in GenBank accession numbers are previously reported *S. polycystum* in Hainan-Guangxi, China by Hu et al. (2018). The value on each node is the mean age (Mya), and the values in each square brackets are the 95% highest posterior probability (HPD) interval.


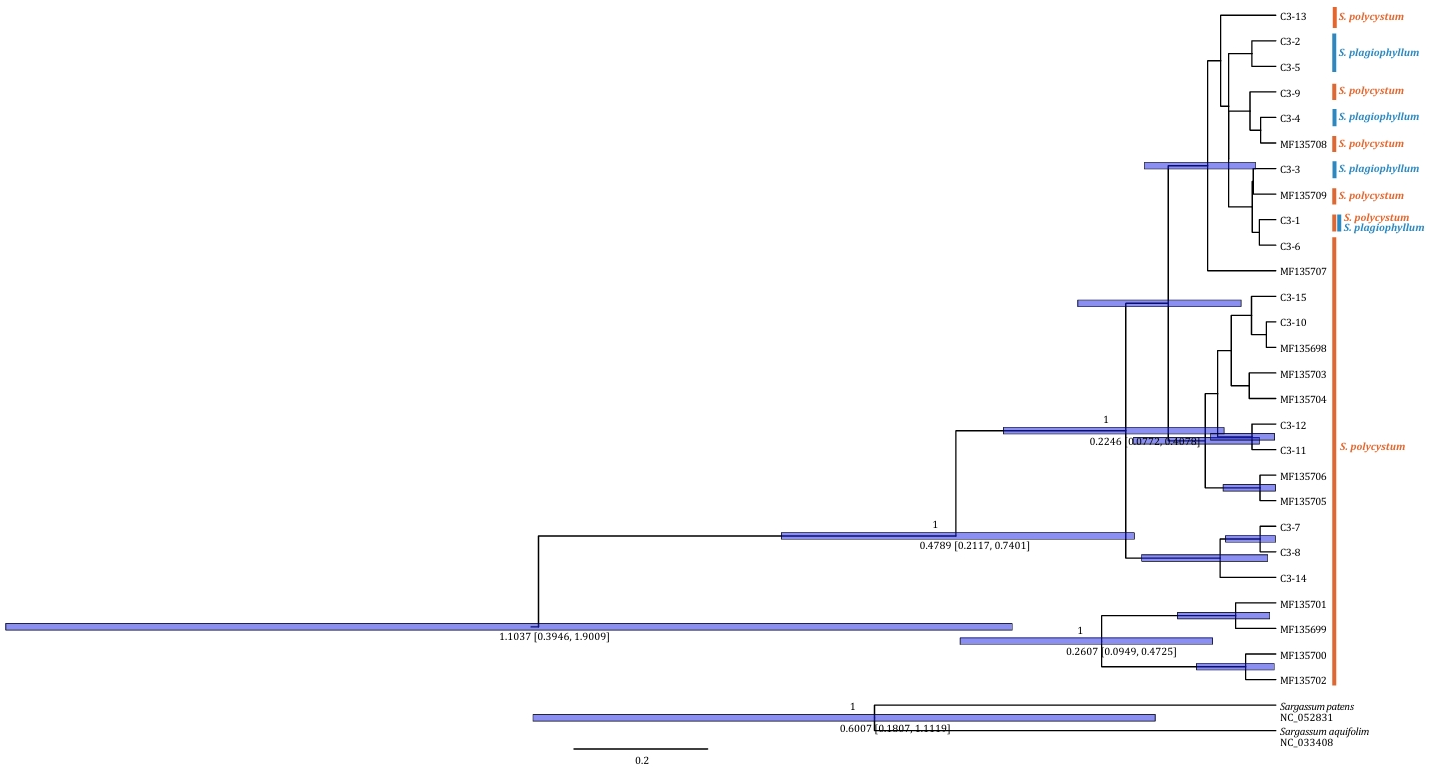


**FIGURE S10** Divergence time estimated among *cox*3 haplotypes of *S. plagiophyllum* and *S. polycystum*. The blue bars represent 95% confidence interval. Unique genotypes showed in GenBank accession numbers are previously reported *S. polycystum* in Hainan-Guangxi, China by Hu et al. (2018). The value on each node is the mean age (Mya), and the values in each square brackets are the 95% highest posterior probability (HPD) interval.


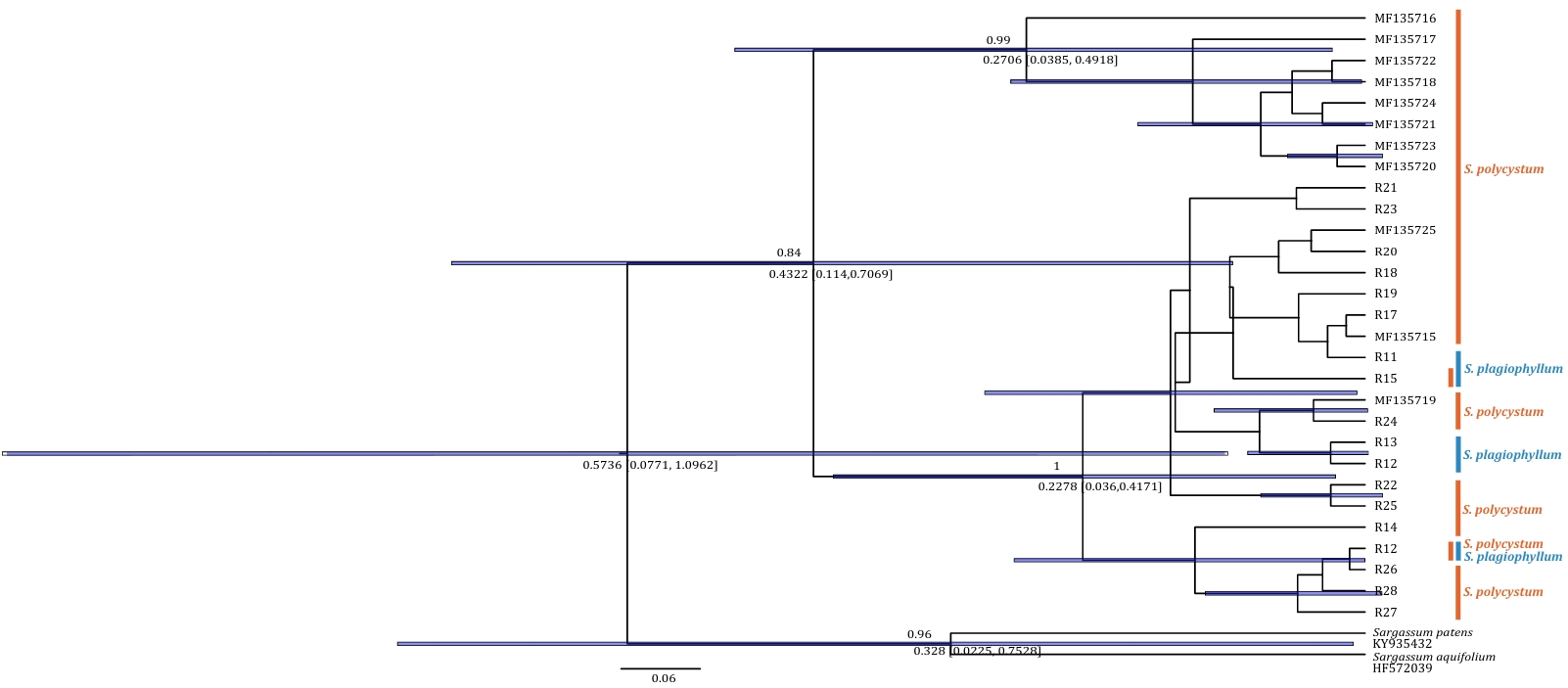


**FIGURE S11** Divergence time estimated among ITS2 ribotypes of *S. plagiophyllum* and *S. polycystum*. The blue bars represent 95% confidence interval. Unique genotypes showed in GenBank accession numbers are previously reported *S. polycystum* in Hainan-Guangxi, China by Hu et al. (2018). The value on each node is the mean age (Mya), and the values in each square brackets are the 95% highest posterior probability (HPD) interval.
